# Screening of Contaminants of Emerging Concern in Surface Water and Wastewater Effluents, Assisted by the Persistency-Mobility-Toxicity Criteria

**DOI:** 10.3390/molecules27123915

**Published:** 2022-06-18

**Authors:** Rosa Montes, Sandra Méndez, Nieves Carro, Julio Cobas, Nelson Alves, Teresa Neuparth, Miguel Machado Santos, José Benito Quintana, Rosario Rodil

**Affiliations:** 1 Institute of Research on Chemical and Biological Analysis (IAQBUS), Universidade de Santiago de Compostela, Constantino Candeira SN, 15782 Santiago de Compostela, Spain; sandramendez.martinez@usc.es (S.M.); jb.quintana@usc.es (J.B.Q.); 2 INTECMAR—Technological Institute for the Monitoring of the Marine Environment in Galicia, Peirao de Vilaxoán S/N, 36611 Vilagarcía de Arousa, Spain; ncarro@intecmar.gal (N.C.); jcobas@intecmar.gal (J.C.); 3 Group of Endocrine Disruptors and Emerging Contaminants, CIMAR/CIIMAR—LA, Interdisciplinary Centre of Marine and Environmental Research, Avenida General Norton de Matos S/N, 4450-208 Matosinhos, Portugal; nalves@ciimar.up.pt (N.A.); tneuparth@ciimar.up.pt (T.N.); santos@ciimar.up.pt (M.M.S.); 4 FCUP—Department of Biology, Faculty of Sciences, University of Porto, Rua Do Campo Alegre, 4169-007 Porto, Portugal

**Keywords:** persistent mobile organic chemicals (PMOCs), screening, quadrupole-time-of-flight (QTOF), passive sampling, water quality

## Abstract

Contaminants of emerging concern (CECs) are compounds of diverse origins that have not been deeply studied in the past which are now accruing growing environmental interest. The NOR-Water project aimed to identify the main CECs and their sources in the water environment of Northern Portugal–Galicia (located in northwest Spain) transnational region. To achieve these goals, a suspect screening analytical methodology based on the use of liquid chromatography coupled to high resolution mass spectrometry (LC-HRMS) was applied to 29 sampling sites in two campaigns. These sampling sites included river and sea water, as well as treated wastewater. The screening was driven by a library of over 3500 compounds, which included 604 compounds prioritized from different relevant lists on the basis of the persistency, mobility, and toxicity criteria. Thus, a total of 343 chemicals could be tentatively identified in the analyzed samples. This list of 343 identified chemicals was submitted to the classification workflow used for prioritization and resulted in 153 chemicals tentatively classified as persistent, mobile, and toxic (PMT) and 23 as very persistent and very mobile (vMvP), pinpointing the relevance of these types of chemicals in the aqueous environment. Pharmaceuticals, such as the antidepressant venlafaxine or the antipsychotic sulpiride, and industrial chemicals, especially high production volume chemicals (HPVC) such as ε-caprolactam, were the groups of compounds that were detected at the highest frequencies.

## 1. Introduction

The interest in contaminants of emerging concern (CECs) in regard to their presence in the environment has been quickly growing. In fact, a large effort has recently been put into mapping their concentration levels in the environment thus contributing to the improvement of risk assessment. In many cases, however, their ecological risk or potential adverse health effects are still unclear [1].

Although some CECs have been met with some regulatory actions, most monitoring programs rely on the application of target methods which cover only a limited number of compounds [2]. As such, in prior years, screening approaches based on liquid chromatography-high resolution mass spectrometry (LC-HRMS) have been developed and applied for the identification of CECs. These approaches allowed researchers to detect a broader range of compounds present in surface water [3,4,5,6]. Among these different approaches, suspect screening has the advantage of being comparatively simpler than non-target strategies. However, its results are highly dependent on the suspect list(s) used. Large suspect lists have the advantage of covering a wider range of compounds at an increased analysis and data treatment cost, particularly if no MS/MS spectra are already available, making them, in a way, similar to non-target strategies. Thus, it is highly recommended to employ suspect lists which either contain MS/MS data or have been developed for a limited set of pollutants which have been previously prioritized based on their properties or use [6,7,8].

One of the most recent prioritization strategies is based on the concepts of persistency (P), mobility (M), and toxicity (T) in the environment [9,10]. This approach was developed as an analogy to the “ persistence, bioaccumulation, and toxicity (PBT) assessment” criteria established under the European chemicals regulation on the registration, evaluation, authorization, and restriction of chemicals (REACH) and the Anex XIII of the Regulation (EC) No 1907/2006, wherein substances are classified according to their persistency (P) based on biodegradation half-life values, bioaccumulation potential (B) based on bioaccumulation factor (BF) on aquatic species, and toxicity (T) based on different toxicity scales [11]. Thus, under REACH legislation, industrial chemicals that are imported or manufactured at more than 10 tons per year need to be evaluated with respect to their PBT (persistent, bioaccumulative, and toxic) and vPvB (very persistent and very bioaccumulative) character. These evaluations feed the list of substances of very high concern (SVHCs) to the Annex XIV of the Regulation (EC) No 1907/2006 [11]. This list needs to be continuously updated, and therefore such classification remains a challenge [12]. Under these criteria, very polar compounds were not a main focus since they usually present low bioaccumulation. However, these substances present a high mobility in the water cycle and, after their emission, they can be transported from the point of emission through soil layers, rivers banks, aquifers, and other natural or even artificial barriers [13]. For that reason, the German Environment Agency (UBA) proposed to name such chemicals in the regulatory context of REACH as persistent, mobile, and toxic (PMT) substances and very persistent and very mobile (vPvM) substances. This placed the focus on their presence in the environment [14], as they pose an equivalent level of concern to PBT/vPvB substances [15]. Thus, considering the large amount of industrial chemicals to be evaluated, some authors have designed prioritization approaches of PMT substances, also called persistent and mobile organic contaminants (PMOCs) based on quantitative structure–activity relationship (QSAR) models and experimental recorded data as a means to reduce the list of substances to be screened [16,17]. Yet, the intrinsically very polar character of PMT/vPvM substances requires newer methods capable of responding to this analytical challenge, such as the use of other retention mechanisms besides reversed-phase LC [13,18,19,20,21]. Furthermore, the combination of different retention mechanisms is highly recommended when a range of polarities is analyzed [6,20,22,23].

The main objective of this work was to investigate the occurrence of CECs in fresh and coastal water, as well as the wastewater effluents discharging into them, in the transnational Galicia (NW Spain)–Northern Portuguese region. To this end, a LC-HRMS suspect screening strategy was employed. The screening was driven by a library of known CECs, with already available MS/MS spectra. This was further complemented by the prioritization of chemicals present in relevant enforcement regulations and recent research on the basis of the abovementioned PMT criteria. The results obtained can be further used to evaluate which chemicals will require a closer monitoring, as well as the significance of such PMT criteria in regard to the pollution of the aquatic environment.

## 2. Materials and Methods

### 2.1. Prioritization of CECs Based on PMT Criteria

A suspect list was constructed by applying the PMT criteria in order to complement the library of CECs with HRMS/MS spectra available in the partners laboratory. In this approach, different sources of compounds were considered (detailed in Table S1) and included (as on September 2019): priority substances in the field of water policy and substances subject to review for possible identification as priority substances or priority hazardous substances in directive 2008/105/EC [24] and 2013/39/EU [25], 1st and 2nd Watch Lists of substances for union-wide monitoring from Commission Implementing Decision (EU) 2015/495 [26] and 2018/840 [27], the 12 performance indicator CECs implemented in Switzerland to ensure the efficiency of the upgraded wastewater treatment plants [28], the candidate list of substances of very high concern (SVHC) by ECHA [29], Annex XIV of REACH substances (list of substances subjected to authorization under EU REACH regulation) [30], OSPAR List of Chemicals for Priority Action [31], EPA Priority Pollutants List under the Clean Water Act [32], NORMAN 2017 monitoring-based prioritization list with a final score above 1.25 [33], lists of REACH registered substances that meet PMT/vMvP criteria as compiled by the German Federal Environmental Agency (UBA) [14] and the Norwegian Geotechnical Institute (NGI) [34], and, finally, compounds detected with high frequency in European surface and drinking water as a result of the PROMOTE project [20]. All these sources are included in electronic Supplementary Material (ESM1), Table S1.

The properties of each of the 818 compounds from this list were screened with different parameters related to persistency, mobility, and toxicity. The octanol/water distribution coefficients (D) at different pH values (4, 7 and 9) were calculated with JChem for Office add-on from Chemaxon [35], and were used as a proxy for mobility. Short-term and long-term toxicity endpoints were obtained from US-EPA ECOSAR Class Program v2.0 [36]. Finally, persistency and toxicity classifications were developed using PBT criteria, the toxic hazard classification with Cramer and the Carcinogenicity (genotox and nongenotox) alerts with ISS, in vitro mutagenicity (Ames test) alerts with ISS endpoints were obtained from the QSAR Toolbox [37]. Moreover, the prioritization results obtained by UBA and NGI for the REACH registered substances were collected [34].

The second step was to establish the criteria for identifying PMT substances. Concerning the mobility criteria, a substance was considered vM if the lowest partition coefficient (log D) in the pH range 4–9 is less than 3.5 and M when log D is between 3.5 and 4.5 [38]. Although the log K_oc_ has also been suggested as a better estimation, this parameter is anyway derived from log D in QSARs, thereby requiring improved models for ionic substances to be implemented in the future [39]. As set for QSAR Toolbox estimations, a substance was considered vP if the biological oxygen demand (BOD) was equal to or lower than 30% and P if BOD ranged between 30 and 40% and/or biodegradation probability was lower than 0.5 (experimental data and BIOWIN 5 and 6 models) [37,40]. Finally, a substance was considered T when the lowest long-term non-observed effect concentration (NOEC) for a marine or freshwater organism was less than 0.01 mg/L, the LC_50_ was less than 1 mg/L, or the substance meets the criteria for classification as carcinogenic, mutagenic, or toxic for reproduction. The compounds classified as Cramer class III were considered as potentially Toxic (Pot T) [37]. The Cramer classification is a well-known approach to estimate the Threshold of Toxicological Concern (TTC) for a chemical substance based on its chemical structure. There are three Cramer classes, with class III representing the most severe toxic hazard [41].

The decision tree used for prioritization is shown in ESM1, Figure S1 and the protocol is explained in further detail in ESM1, Text S3.

Based on this assessment, five different final groups were created:

- vPvM: all the sources indicate that these compounds are vP and vM. T was not investigated, since the fact of being vPvM indicate that they are already of concern (Figure S1)
- PMT: all the sources indicate that these compounds are P, M, and T.
- PM-Pot T: all sources indicates that these compounds are P and M and were classified as Pot T using the Cramer classification scheme.
- Potential PMT: non-concordant results were reported and a conclusion for P and/or M and/or T was obtained.
- Not PMT: all sources indicate that these compounds are either not P and/or not M and/or not T.

For screening purposes, Not PMT chemicals as well as substances that are not amenable to analysis with LC-MS (such as inorganic ions) were excluded from the final suspect list (see below and ESM3).

### 2.2. Samples

A total of 29 sampling sites were analyzed, including 16 points located in 4 rivers (Miño, Limia, Ave and Cavado), 8 points located in estuarine areas (Ría de Vigo), and 5 wastewater treatment plants (WWTPs) in Galicia (NW Spain) and North-Portugal. The exact locations of surface water sampling points are given in ESM1, Table S2. WWTPs have been codified and only general characteristics are provided due to confidentiality agreements. In the case of river and estuarine/sea water, two different sampling strategies have been used: grab sampling and polar organic chemical integrative samplers (POCIS), in the last case with a deployment time of 1 (river) or 2 (estuarine/sea) weeks [6]. In the case of WWTPs, only treated (effluent) wastewater was analyzed, and 24 h composite samples were collected. Two different sampling campaigns were performed, in January–February and June–July 2020.

### 2.3. Screening Method

Grab river or sea water samples (200 mL) and treated wastewater (100 mL) were filtered and processed using mixed-mode solid-phase extraction (MM-SPE) following the protocol described by Montes et al. [19]. POCIS samplers were prepared using 500 mg of OASIS HLB sorbent and were desorbed after the sampling period, following the protocol described by Castro et al. [6]. In both cases, 0.5 mL of concentrated methanolic extract were obtained. These extracts were injected in an Agilent 1290 LC coupled with an Agilent 6550 QTOF system using various chromatographic and instrumental conditions adapted from [19,42]. Reversed-phase (RP) and mixed-mode (MM) chromatography were used in order to expand the polarity range of the possible compounds detected. A data-independent, all ions, acquisition workflow (at 20 V of collision energy in the high energy experiment) was used, and all samples were injected in both positive and negative ionization modes. More detailed information is provided in ESM1, text S1.

A mixture containing 18 isotopically labelled CECs (ESM1, Text S2) was prepared and added to each sample (200 ng/L level). The chromatographic peak shape and retention time of those compounds were evaluated in each injection in order to verify absence of chromatographic (i.e., retention time stability and peak shape) and MS (mass accuracy and sensitivity) issues during the entire protocol. Variations lower than 5% in retention times were observed for those analogs in all the injections performed.

Finally, field blanks were carried out and the compounds detected in blank experiments were only reported if signal intensities in samples were 3 times higher than those observed in blanks.

### 2.4. Data Analysis

The obtained chromatograms were processed using the MassHunter Qualitative software V10.0 from Agilent Technologies (Santa Clara, CA, US) and a suspect screening search based on the algorithm “find by formula” and two compound databases (PCDL) were performed. The first one (Lib A) was an accurate mass MS/MS spectral library containing 3322 entities created by combining four commercial libraries supplied by Agilent (ForTox PCDL, Water PCDL, Pesticides PCDL and VetDrugs PCDL) and an in-house empirical MS/MS library (ESM2). Among the chemicals included in Lib A, 294 were considered in the prioritization strategy being 250 analytes finally prioritized by the PMT criteria (ESM2, ESM3). The second one (Lib B) was a database of formulas and compound identifiers (no MS/MS) which was created with the remaining LC-ESI amenable prioritized substances (ESM2) not contained in Lib A. More detailed information on the data analysis workflow is provided in ESM1, Text S1.

## 3. Results and Discussion

### 3.1. Prioritization

The selected sources of information (ESM1, Table S1) resulted in a list containing 818 compounds. Once the PMT properties of these 818 chemicals were collected, the assessment procedure for prioritization was applied (ESM1, Figure S1 and ESM3).

**Figure 1:**
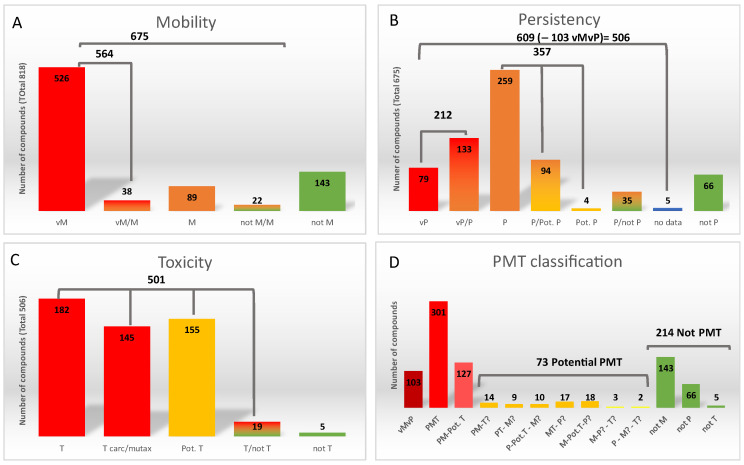
Results of the prioritization strategy. (**A**) Mobility classification, (**B**) persistency classification, (**C**) toxicity classification, (**D**) PMT criteria classification.

First, regarding mobility, of the 818 compounds 526 were classified as very mobile (vM) (log D < 3.5), 89 as mobile (M) (3.5 < log D < 4.5), and 143 compounds were classified as non-mobile (not M). The not M compounds were not further considered (Figure 1A and ESM3). The remaining 60 compounds had an ambiguous classification depending on the source of log D. Thus, 38 compounds were added to the vM class using the worst-case scenario (M or vM, depending on source). On the other hand, for 22 compounds the disagreement was between non-mobile (not M) and M and they were considered as “mobility classification uncertain” (M?).

Thus, 675 compounds were evaluated for the following criterium: persistency. From these compounds, 79 compounds were classified as very persistent (vP), 259 as persistent (P), and 66 as non-persistent (not P). These not P compounds were discarded in the following evaluations. For the remaining 271 compounds, classification varied according to the source and whether there were insufficient data to classify them (Figure 1B, and ESM3). Considering these divergences, those compounds with an uncertain classification (between vP/P, vP/PotP, P/Pot.P or Pot.P) were classified according to the worst scenario (vP or P). Therefore, 133 and 98 compounds were added to the of vP and P groups, respectively. The remaining 40 compounds were classified as “persistency classification uncertain” (P?). At this step, 103 compounds were directly prioritized since they fall into the vMvP category and were not investigated for toxicity.

The toxicity of the remaining 506 compounds (those being P/P? and M/M?) was investigated by considering first the acute and chronic toxicity for freshwater and saltwater organisms, then potential mutagenicity or carcinogenicity, and finally the Cramer classification as detailed in ESM1, Text S3. This resulted into 327 compounds being sorted as T (because of acute/chronic toxicity or mutagenic/carcinogenic character), 155 as potentially toxic (Pot T, Cramer class III), 5 as non-toxic, and 19 resulting into non-conclusive toxicity (Figure 1C, and ESM3).

In summary, the final classification using 5 categories (vPvM, PMT, PM Pot T, Potential PMT and not PMT) is detailed in the last column (AP column) of ESM3. The prioritized chemicals list consists of 103 vPvM chemicals, 301 PMT chemicals, and 127 PM-Pot T chemicals (Figure 1D). Moreover, 68 chemicals that fulfilled two of the criteria but had unconclusive data for the third one and 5 compounds that fulfilled one criterium but had unconclusive data for the remaining two, were considered as Potential PMT and also prioritized for the suspect list. Thus, 604 compounds were considered as priority for further studies (ESM3).

This final list of PMT and PM-Pot T contains already known CECs, such as the herbicide terbutryn or the pharmaceutical trimethoprim, but also other chemicals that are less studied as contaminants at this moment and whose presence in the environment should be studied due to their properties, such as cyanuric acid, naphthalene sulfonic acid, or metformin. When the final classification of substances is analyzed according to the sources of information (Figure S2), it is observed that a large number of the substances that are currently monitored following water legislation [24,25] or the SVHC list [29] would not meet the PMT criteria. However, there is a trend towards the inclusion of this type of substances in new prioritization strategies carried out by other authors [14,28,34] and the recent Watch Lists [26,27], where up to 90% of the substances included are vMvP, PMT, or PM Pot T (Figure S2).

### 3.2. Sample Preparation and Analytical Considerations

The sample preparation protocol was designed to cover compounds with a wide range of physical and chemical properties. In the case of passive sampling the nature of polyethersulfone (PES) membranes and the OASIS HLB sorbent, whose retention is mainly based on a reversed-phase mechanism, allowed the recovery of low and intermediate polarity analytes. For most polar and ionizable compounds, the use of mixed-mode SPE phases, is more appropriate. Thus, as explored in previous works [19], the use of OASIS WCX and OASIS WAX sorbents allows the retention through cationic and anionic exchange besides reversed-phase mechanism. This increased retention capability helps broaden the range of retained compounds. Considering the possible charge state or the most likely tendency to ionize in both polarities in the ESI source, the OASIS WAX extracts were analyzed for the ESI (−) ionizable compounds, the OASIS WCX extracts for the ESI (+) ionizable compounds, and the OASIS HLB sorbent from the POCIS samplers for all the analytes.

As in the case of extraction protocol, to cover the highest number of analytes, different chromatographic modes were tested, including supercritical fluid chromatography (SFC), mixed-mode chromatography (MMLC), and reversed-phase chromatography (RPLC). As suggested in other works [3,6], SFC should provide good results in terms of the number of chemicals that could be detected; however, when the extracts of OASIS WCX and WAX cartridges were injected, many problems of clogging and overpressure were detected, probably due to salts precipitation under supercritical conditions. Thus, SFC was discarded in this work. All the extracts obtained from POCIS and SPE protocols were injected using both MMLC and RPLC. The results, in terms of identified compounds, obtained after data treatment of chromatograms from both chromatographic approaches—MMLC and RPLC—were considered together. This was proposed as the best combination when SFC is unavailable [6]. Although hydrophilic interaction chromatography (HILIC) was also explored by other authors [3,22], it was not included in this work.

### 3.3. Screening Results

**Figure 2:**
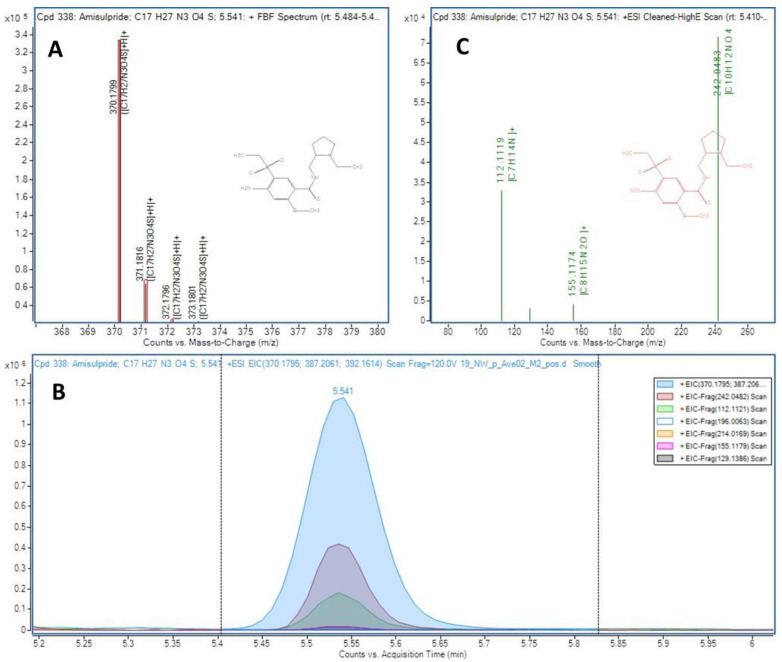
Identification of amisulpride in sample A2. (**A**) Match for the [M+H]^+^ isotopic distribution (theoretical values as red boxes), (**B**) Extracted ion chromatograms for the co-eluting fragment ions in the high energy channel (20 V) and (**C**) Composite MS/MS spectrum generated by the software with the fragment ions matching the library information.

The summary of results obtained in the two sampling campaigns and the 29 sampling sites, in terms of number of compounds detected, is shown in ESM4 (the detailed detection data of each sampling point is also provided in ESM5). Thus, 343 analytes could be identified by coincidence of MS/MS fragment ions coeluting in the high energy channel and/or by MS Score in the low energy channel, i.e., levels 2a and 3 of confirmation according to the levels proposed by Shymansky et al. [43] as explained in ESM1, text S1. In the case of analytes confirmed as level 3 (167), more than one candidate had coincident fragments coeluting in the high energy MS experiment (20 V). Thus, the candidate with the highest number of coincident fragments was proposed. In the case of the 169 compounds identified as level 2a, the match between the coeluting fragments and the library spectrum was unambiguous, with at least two coincident fragments. An example of a compound (amisulpride) identified as level 2a using this data-independent workflow is presented in Figure 2, showing the coeluting peaks in the extracted ion chromatograms (low and high energy channels)of several fragment ions which match the spectra contained in the Lib A for this compound. For the 7 compounds identified using the Lib B, where no MS/MS spectra were originally available, experimental MS/MS spectra were obtained with a further target MS/MS re-injection of the sample and were manually compared with those recorded in open-source databases (such as Massbank and Mzcloud) and classified as level 2a (ESM4). An example of a compound identified using Lib B database is shown in Figure S3. The 6:2 fluorotelomer sulfonate (6:2 FTSA) presented a MS Score higher than 95 and the MS/MS spectrum matched the recorded in MzCloud (reference number 8374) for the molecular ion and two fragment ions (Figure S3).

From the 343 identified chemicals, 112 had already been prioritized as vMvP/PMT substances (ESM3). The remaining 231 chemicals were submitted to the entire decision tree for PMT classification. This resulted in 153 chemicals classified as PMT, 23 as vMvP, 90 as PM-Pot T, and 1 as Potential PMT. Thus, this means that over 75% of the identified substances in the studied area were persistent and mobile substances. Furthermore, 65 substances (19% of the total), despite predicted to be non-persistent, were found in the samples, likely due to their high production/usage volume and incomplete removal at WWTPs.

Regarding the frequency of appearance, from the 343 identified substances, 37 chemicals were found in more than 50% of analyzed samples in at least one campaign. Most of these substances were pharmaceuticals with a high degree of medical prescription, but there are also other industrial high-volume production chemicals such as sulfonates or perfluorinated compounds. The 10 topmost frequently detected compounds are discussed in the next section.

**Figure 3:**
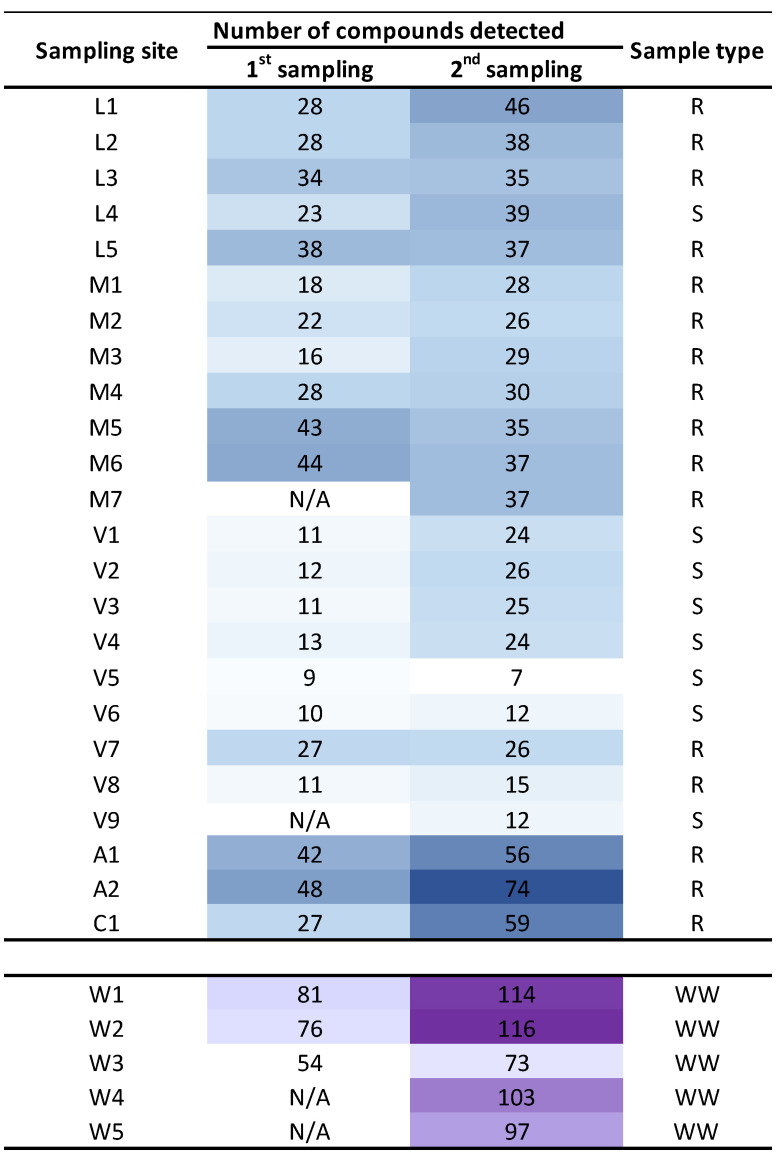
Heat map of number of compounds identified per sampling site and campaign. Sample type code: river water (R), sea and transitional water (S) wastewater (WW). First sampling was held during January–February 2020 and the second sampling was held during June–July 2020. No POCIS sampling is considered. N/A (not analyzed).

Figure 3 shows a heatmap with the level of pollution per sampling site, considering the number of chemicals detected. In this case, only grab samples data (or composite samples in WWTP) are included, since at some sampling sites the passive sampling was not possible (ESM1, Table S2). As expected, WWTP treated water samples were the most polluted ones, together with those sampling points located in rivers near the WWTP emissaries or downstream of population settlements (such as L1 and A2, Figure 3). On the other hand, the sea water samples from the outer area of the Ría de Vigo were the less contaminated ones, possibly due to dilution (V5 and V6, Figure 3).

When the seasonal variation was analyzed, a statistically significant increase (*t*-test for paired samples presented in Figure 3, *t(25)* = −5.9, *p* = 3 × 10^−6^) was observed in summer (dry season) when the dilution is lower due to a lower water flow in the rivers course. However, this difference is lower for sea or transitional waters (V5, V6, V8) or in the widest part of the rivers (L3, L5, M5, M6) where the flow undergoes a negligible variation.

### 3.4. Overview on the Most Frequently Detected Contaminants

Table 1 shows a summary containing the 10 most frequently detected chemicals, i.e., those presenting a frequency of detection higher than 80% in at least one of the sampling campaigns.

#### 3.4.1. 1,3-Di-O-Tolylguanidine (DTG) and 1,3-Diphenylguanidine (DPG)

DTG and DPG are substances used in the manufacturing of rubber and other polymers with a high production volume in Europe. Both compounds were reported to occur in different water samples across Europe [20]. DPG was recently reported to occur in run-off water from roads [44,45], pointing to tire crumb rubber as a potential source of this compound in surface water. Although both DTG and DPG have been observed to be transformed by chlorination [46], or, in the case of DPG, also through solar radiation [5], their presence in the environment or ecotoxicological effects have scarcely been studied.

#### 3.4.2. Dodecylbenzene Sulfonate (C12-LAS)

This chemical is a widely used surfactant, whose presence in the environment, especially its occurrence and transformation in wastewater treatment plants and subsequent release to surface waters, has been previously reported [47]. C12-LAS is classified as PM Pot T. This classification as persistent, as based on the PBT assessment of the QSAR toolbox, appears to be in contradiction to the efficient removal of this compound even after WWTP treatments [48]. However, the P evaluation refers to natural processes occurring in surface waters and not specifically to the treatment conditions of degradation in WWTPs. Regarding the C12-LAS toxicity, this compound was finally classified as Pot T, being a Cramer class III compound. However, when analyzing the toxicity data, the lowest LC_50_ and NOEC are 1.2 and 0.04 mg/L, respectively, as reported for *Daphnia magna* being close to the selected toxicity threshold of 1 and 0.01 mg/L for LC_50_ and NOEC, respectively (ESM3). In addition, the literature on the toxicity evaluation of linear alkylbenzene sulphonates is vast, stating that they could be considered toxic for some organisms. This literature on toxicity was not considered in our decision tree, increasing the concern about C12-LAS reported in this work [49]. It should be noted that the decrease in the frequency of appearance observed in the June–July campaign responds to an unexpected increase in the signal of laboratory blanks during this sampling period due to the wide usage rate of these surfactants derived from the COVID19 pandemic situation.

#### 3.4.3. ε-Caprolactam

ε-caprolactam is a reagent used in nylon and other materials manufacturing, with more than 1 million tons/year produced in Europe. In these sampling campaigns, ε-caprolactam appeared in more than 80% of samples and was also reported as water pollutant in several recent studies [20,50]. This compound was classified as not P; however, it is vM and also considered T [14]. The observed high frequency of detection reflect its high usage rate and easy release into the environment which results into a continuous input, despite its degradability. Therefore, its inclusion in future studies is highly recommended.

#### 3.4.4. 4-Nitrophenol (4-NP), Methyl 4-Hydroxybenzoate (Methyl Paraben), and Tributoxyethyl Phosphate (TBEP)

These three compounds are well-known substances. Of these, 4-NP, which is classified as PMT, has several sources and uses such as pesticide and as intermediate or precursor in different industrial processes, and has been thoroughly reported as a water contaminant [42,51]. Following the criteria applied in this work, methyl paraben should be classified as not P, which is supported by some experimental investigations [52], but the results obtained point to a similar conclusion to ε-caprolactam. Although some authors have recently suggested a possible natural origin of this compound [53], the ubiquity shown and the literature on the toxicological effects [54] make it a candidate for future monitoring studies. On the other hand, TBEP is also a well-known CEC classified as PMT, and its presence in the environment and ecotoxicological and human health effects have been already studied [55].

#### 3.4.5. Venlafaxine, o-Desmethylvenlafaxine, and Sulpiride

Venlafaxine is one of the most prescribed antidepressants in Spain [56], while o-desmethylvenlafaxine is its main human metabolite, though it has also been prescribed as an antidepressant since 2014, albeit to a lesser extent than venlafaxine [57]. Both are PMT substances according to the criteria applied and have been frequently found in wastewater effluent samples and connected surface waters [4]. Both substances have been included in the 2020 Decision implementing the 3rd surface water Watch List [58]. Their relevance is stressed in this work since they were also found in estuarine environments. Finally, the most frequently detected chemical when considering both campaigns was sulpiride, an antipsychotic drug [57]. After the application of the same criteria used for prioritization, this drug was also classified as PMT. The 2 other antipsychotic drugs of the same class found in this screening, amisulpride and tiapride, would also be classified as PMT, although they were detected less frequently. This lower frequency of detection in samples could also be attributed to a lower defined daily dose (DDD) for the psychosis treatment of the latter compounds (0.4 g) compared with recommended DDD of sulpiride (0.8 g) [59]. Also, sulpiride has an additional pharmacological use as antidopaminergic in dizziness disease treatment. Finally, a low removal efficiency in WWTPs, together with high excretion rate (around 70%) and low bioaccumulation was reported for this compound [60], may explain the high frequency of detection in water.

## 4. Conclusions

We have performed a comprehensive screening of CECs in Spanish and Portuguese surface water and effluents (as a major contributor to surface water CECs). The screening was supported by a prioritization strategy of chemicals from relevant enforcement and recent research. By screening for PMT properties, a list of 604 chemicals was compiled and summed to an accurate mass MS/MS library of over 3300 chemicals. Real samples were then submitted to a screening methodology based on the use of LC-HRMS and two SPE-based sample treatment approaches. Passive sampling was also performed in some of the sampling points. One hundred and twelve of the compounds derived from the prioritization study were identified in the samples. Furthermore, over 50% of the 343 pollutants identified in this study were tentatively classify as PMT or vMvP substances. Thus, the list of the most frequently detected substances can be used as a priority for future targeted studies.

## Figures and Tables

**Table 1:** Topmost frequently detected compounds.

Name	Cas	Production (Tons/Year) (1)	Main Uses	LogD pH7.4 (2)	Freq (%) March	Freq (%) July	Classification (3)
1,3-di-o-tolylguanidine (DTG)	97-39-2	100–1000	Vulcanization reagent	2.27	100	66	PM-Pot T
1,3-diphenylguanidine (DPG)	102-06-7	1000–10,000	Vulcanization reagent	2.89	38	80	PM-Pot T
Dodecylbenzenesulfonic acid (C12-LAS)	18777-53-2	1000–10,000 *	Manufacture washing–cleaning products	4.02	100	10	PM-Pot T
ε-caprolactam	105-60-2	1,000,000–10,000,000	Manufacture polymers, textiles, coatings, soaps	0.31	100	80	not P
Methyl 4-hydroxybenzoate (Methyl paraben)	99-76-3	1000–10,000	Cosmetical and personal care products	1.64	85	69	not P
Tributoxyethyl phosphate (TBEP)	78-51-3	1000–10,000	Manufacture polymers and textiles	3.94	73	100	PMT
4-Nitrophenol (4NP)	100-02-7	Unavailable	Chemicals manufacturing	1.12	46	90	PMT
Venlafaxine	93413-69-5	Unavailable	Pharmaceutical	1.22	69	80	PMT
O-desmethylvenlafaxine	93413-62-8	Unavailable	Pharmaceutical metabolite	1.07	62	90	PMT
Sulpiride	15676-16-1	Unavailable	Pharmaceutical	−0.7	81	100	PMT

(1) Information from ECHA website (2) Calculated with JChem for excel (3) According to the criteria used in this study. * Information for sodium salt, but at least other 3 formulations produced.

## Supplementary Materials

The following supporting information can be downloaded at: https://www.mdpi.com/article/10.3390/molecules27123915/s1, Text S1: Determination parameters and data treatment workflow applied, Text S2: Information on mass labelled analytes used to check for method reliability, Text S3: Detailed protocol used for compound prioritization, Table S1: Sources of information for the selection of target compounds, Table S2: Sampling sites information, Figure S1: Decision tree for prioritization protocol, Figure S2: Classification of the substances contained in different sources of information according to this work’s criteria, Figure S3: Identification of 6:2FTSA in sample A2. (A) Match for the [M − H]- isotopic distribution (theoretical values as red boxes), (B) Extracted ion chromatogram of the [M − H] − (m/z 426.9679) and (C) experimental MS/MS spectrum at 20 V of collision energy, to be compared with recorded in MzCloud (reference number 8374), ESM2: Compounds contained in the PCDL databases (Lib A and Lib B) used in the suspect screening approach, ESM3: Compounds included in the prioritization strategy, their properties and final classification according to the established PMT criteria, ESM4: Compounds identified in the suspect screening approach, frequency of detection in each sampling campaign and classification according to the PMT criteria, ESM5: Details on the compounds identified in the suspect screening approach by sampling site in both sampling campaigns.
