# Supplementary material for: Screening of Contaminants of Emerging Concern in Surface Water and Wastewater Effluents, Assisted by the Persistency-Mobility-Toxicity Criteria
Source: Molecules. 2022 Jun 18;27(12):3915. doi: 10.3390/molecules27123915 (PMC9227630; doi:10.3390/molecules27123915)
Supplement: Supplementary file 1 [file molecules-27-03915-s001.zip › ESM1.pdf]

ELECTRONIC SUPPLEMENTARY MATERIAL TO:

"Screening of contaminants of emerging concern in surface water and wastewater effluents, assisted by the persistency-mobility-toxicity criteria"

Rosa Montes [1,*], Sandra Méndez [1], Nieves Carro [2], Julio Cobas [2], Nelson Alves [3,4], Teresa Neuparth [3],
Miguel Machado Santos [3,4], José Benito Quintana [1] and Rosario Rodil [1,*]

[1] Institute of Research on Chemical and Biological Analysis (IAQBUS), Universidade de Santiago de Compostela, Constantino Candeira SN, 15782 Santiago de Compostela, Spain; sandramendez.martinez@usc.es (S.M.); jb.quintana@usc.es (J.B.Q.)
[2] INTECMAR- Technological Institute for the Monitoring of the Marine Environment in Galicia, Peirao de Vilaxoán S/N, 36611 Vilagarcía de Arousa, Spain; ncarro@intecmar.gal (N.C.); jcobas@intecmar.gal (J.C.)
[3] CIMAR/CIIMAR—LA, Interdisciplinary Centre of Marine and Environmental Research,
Group of Endocrine Disruptors and Emerging Contaminants, Avenida General Norton de Matos S/N,
4450-208 Matosinhos, Portugal; nalves@ciimar.up.pt (N.A.); tneuparth@ciimar.up.pt (T.N.);
santos@ciimar.up.pt (M.M.S.)
[4] FCUP-Department of Biology, Faculty of Sciences, University of Porto, Rua Do Campo Alegre,
4169-007 Porto, Portugal
* Correspondence: rosamaria.montes@usc.es (R.M.); rosario.rodil@usc.es (R.R.)

ESM2: Compounds contained in the PCDL databases (Lib A and Lib B) used in the suspect screening approach (independent file)

ESM3: Compounds included in the prioritization strategy, their properties and final classification according to the established PMT criteria (independent file)

ESM4: Compounds identified in the suspect screening approach, frequency of detection in each sampling campaign and classification according to the PMT criteria (independent file)

ESM5: Details on the compounds identified in the suspect screening approach by sampling site in both sampling campaigns (independent file)

Text S1: Determination parameters and data treatment workflow applied

Text S2: Information on mass labelled analytes used to check for method reliability

Text S3: Detailed protocol used for compound prioritization

Table S1: Sources of information for the selection of target compounds

Table S2: Sampling sites information

Figure S1: Decision tree for prioritization protocol

Figure S2: Classification of the substances contained in different sources of information according to this work's criteria

Figure S3: Identification of 6:2FTSA in sample A2. A) Match for the $[M\text{-}H]^-$ isotopic distribution (theoretical values as red boxes), B) Extracted ion chromatogram of the $[M\text{-}H]^-$ (*m/z* 426.9679) and C) experimental MS/MS spectrum at 20 V of collision energy, to be compared with recorded in MzCloud (reference number 8374)

Text S1: Determination parameters and data treatment workflow applied

Instrumental conditions

Chromatographic separation was performed on an Agilent Technologies (Wilmington, DE, USA) 1290 Infinity II series using a ZORBAX Extend-C18 (Agilent) 2.1 × 50 mm, 1.8 µm column connected to a Supelco ColumnSaver 0.5 µm Precolumn Filter (Supelco, Bellefonte, PA, USA). The temperature at the column was fixed at 35 °C and a 2 µL aliquot of the sample was injected. As mobile phases, methanol (A) and water (B) containing 0.1% formic acid (pH 2.7) for injections performed in ESI positive mode and 5 mM of NH_4CH_3COOH (pH 6) for injections performed in ESI negative mode, were used at flow rate of 0.4 mL/min. The gradient elution started with 98% A, increasing to 100% B in 22 min, held for 4 min. Subsequently, it returned to initial conditions (98% A) and was held for 4 min for column back-conditioning. Thus, the total chromatographic run lasted 30 min.

The UHPLC system was coupled to an Agilent 6550 iFunnel QTOF LC/MS system. The QTOF was furnished with a Dual Agilent Jet Stream electrospray (ESI) ion source. The ESI interface was operated either in positive or negative modes (in different injections) and the needle voltage of the ESI was fixed at 3500 V. Nitrogen was used as nebulizing (30 psi) and drying gas (200 °C, 12 L/min) in the ESI source, and also as collision gas in the MS/MS experiments. Thus, 2 injections were performed per sample, in either positive or negative polarity.

Instrument control and data acquisition were performed with Agilent MassHunter Workstation software B.10.00. Data acquisition was performed by an All Ions (data-independent acquisition, DIA) method, with two alternating collision energies of 0 (low energy), where little fragmentation is expected, and 20 V (high energy) where fragments are expected. Hence, in this mode, assignations of potential fragments to precursor ions are based on the coelution of such potential fragments in the 20 V channel with the precursor ion in the 0 V channel. The scanned range was 40-1100 m/z. A reference calibration solution, supplied by Agilent, was continuously sprayed in the source during the chromatographic run, in order to continuously recalibrate the m/z axis. The purine and HP-0921 (hexakis (1H, 1H, 3H-tetrafluoropropoxy) phosphazine) $[M + H]^+$ ions, 121.050873 m/z and 922.009798 m/z, respectively, were employed in positive mode for this purpose. The trifluoracetic acid $[M\text{-}H]^-$ ion (112.985587 m/z) and the formate (966.000725 m/z) and trifluoroacetate (1033.988109 m/z) adducts of HP-0921 were used in negative mode.

Data analysis

Data files were processed using the Qualitative Workflows of Agilent MassHunter Workstation software B.10.00. The All Ions data files were processed using the search algorithm Find by Formula, where the software searches for possible ions matching with the empirical formula considering $[M+H]^+$, $[M+NH_4]^+$, $[M+Na]^+$ ions in positive mode and only $[M\text{-}H]^-$ in the negative mode (score>85% and mass error lower than 5 ppm) in the low energy channel. Two PCDL type databases provided in file ESM2, Lib A, containing MS/MS spectra of ca. 3200 chemicals, including pharmaceuticals, pesticides and other emerging pollutants and Lib B containing formula and exact mass of 209 compounds, from the prioritization strategy, that were not included in Lib A, which means that MS/MS spectra were not originally available for them. Note that the MS score is a combination of mass error against theoretical mass, isotopic profile match

and spacing among those isotopes. When a match is found, and if the library contains MS/MS spectra (case of Lib A), up to 7 product ions from the MS/MS library spectrum are extracted as chromatograms from the high energy channel, generating a unique Co-elution Score, which indicates confidence of the correlation between the precursor and the product ions by peak shape, peak width, and retention time. Thus, for Lib A, a compound was considered as identified if the MS score was higher than 85 and there was a co-elution (Score higher than 80) for at least 2 fragment ions compared with the spectrum recorded in the PCDL (Lib A). When only one candidate fulfilled the identification criteria this was assigned confirmation level was 2a. If there were various candidates fulfilling the identification criteria, that one presenting higher number of co-eluting fragment ions or the highest MS Score was selected and was assigned confirmation level 3. On the other hand, when no spectral information was originally available (case of Lib B), candidates with MS Score higher than 85 and mass error lower than 5 ppm were initially set as level 4. Subsequently their actual MS/MS spectra (at 10, 20 and 40 V) was acquired in target MS/MS experiments and their spectra compared to open-source libraries (Massbank and mzCloud), being re-classified as level 2a if there were at least to coincident product ions.

All the confirmation levels mentioned here are based on Schymanski et al. [43].

Text S2: Information on mass labelled analytes used to check for method reliability

Mass labelled compounds information

A mixture containing 3000 ng mL $^{-1}$ of the following compounds was added to the samples (200 ng L^{-1} level referring to the samples): Amphetamine-d_6, benzoylecgonine-d_3, cocaine-d_3, diazepam-d_5, fluoxetine-d_6, ritalinic acid-d_{10}, sertraline-d_3, temazepam-d_5 and venlafaxine-d_6 were supplied by Cerilliant (Round Rock, TX, USA) as 100 µg mL^{-1} solutions in MeOH; tri-n-butyl phosphate (TnBP)-d_{27}, tris(2-chloroisopropyl) phosphate (TCPP)-d_{18} and $^{13}C_8$-perfluorooctane sulfonic acid ($^{13}C_8$-PFOS) were supplied by Wellington Laboratories (Guelph, Ontario, Canada); Cyprodinil-d_5 and $^{13}C_6$ metalaxyl were supplied by Riedel-de Haën (Seelze, Germany); Methylparaben-d_4 was supplied by CDN Isotopes (Quebec, Canada); Atenolol-d_7 and irbesartan-d_4 were supplied by Sigma-Aldrich (St. Louis, MO, USA); Metformin-d_6 was supplied by TRC Canada (Ontario, Canada).

Text S3: Detailed protocol used for compound prioritization

The first step in the prioritization strategy was the assessment of the mobility based on the log D in the pH range 4-9 using the data obtained from JChem and on the classification provided in the Reports of the German Environment Agency (UBA) [14]:

- Very Mobile (vM): Those compounds with the minimum JChem predicted log D in the pH range 4-9 below 3.5 and classified (or not considered) by UBA as vM.
- Mobile (M): Those compounds with the minimum JChem predicted log D in the pH range 4-9 between 3.5 and 4.5 and classified (or not considered) by UBA as M.
- Mobility classification uncertain (M?): Those compounds with non-concordant results between the two sources.

The second step was the assessment of persistency which was applied to those compounds that have been classified as vM, M or M?. Persistency classification was performed based on the QSAR Toolbox classification using PBT criteria and on the classification provided in the Reports of the German Environment Agency (UBA) [(A)]. Thus, the chemicals were classified as:

- Very Persistent (vP): Those compounds classified as vP by both sources, or only considered in one of them.
- Persistent (P): Those compounds classified as P by both sources, or only considered in one of them.
- Persistency classification uncertain (P?): Those compounds with non-concordant results between the two sources.

The last step was the assessment of toxicity. Only substances classified as vP (excepting those being vPvM), P or P? were evaluated for toxicity. Toxicity classification was performed in three steps:

1- Toxicity classification based on: the NOEC and/or LC_{50} reported by ECOSAR, the QSAR Toolbox classification using PBT criteria, and the classification provided in the UBA Reports. Those compounds classified as Toxic (T) for all the three sources (or at least all the sources that provide data for them) are considered Toxic (T).
2- Those substances with non-concordant results or not classified as T in the previous step were assessed for carcinogenic, mutagenic, or toxic for reproduction based on the QSAR Toolbox classification and UBA Reports. Those compounds which present carcinogenic or mutagenic effect were considered as Toxic (T).
3- For those substances which did not meet the previous T criteria, the chemical structure of the substance was screened using the Cramer classification scheme from the QSAR Toolbox and UBA Reports [(A)]. Those compounds with Class III classification were considered "Potential T" (Pot. T).

Table S1. Sources of information for the selection of target compounds.

Source	Number of chemicals	Reference
Directive 2008/105/EC	44	24
Directive 2013/39/EU	50	25
1st watch list of substances	17	26
2nd watch list of substances	15	27
SWISS List on WWTP removal	12	28
ECHA Substances of Very High Concern	122	29
Annex XIV of REACH substances	23	30
OSPAR List of Chemicals for Priority Action	38	31
EPA Priority Pollutants List	97	32
The criteria for identifying Persistent, Mobile and Toxic (PMT) substances and very Persistent and very Mobile (vPvM) substances under EU REACH Regulation	328	14
Monitoring based Priorization NORMAN2017	195	33
Main PMOCs identified in PROMOTE project	17	20
REACH: Improvement of guidance and methods for the identification and assessment of PMT/vPvM substances. German Environment Agency	260	34

Table S2: Sampling sites information

Sample code	Type	Coordinates X (UTM ETRS89/H29)	Coordinates Y (UTM ETRS89/H29)	Grab sample	POCIS
L1	River	603551.76	4657145.57	x	x
L2	River	592488.56	4652796.92	x	x
L3	River	531418.06	4623146.44	x	x
L4	Sea	514824.17	4615499.98	x	x (a)
L5	River	554704.79	4629452.52		
A1	River	526638.85	4577839.33	x	x
A2	River	522809.75	4578452.3	x	x
C1	River	538305.97	4599734.97	x	x (b)
M1	River	594126.97	4689328.78	x	x (b)
M2	River	587124.19	4688072.07	x	x
M3	River	582689.17	4688613.71	x	x (b)
M4	River	529492.11	4654775.63	x	x
M5	River	538502.71	4656342.26	x	X
M6	River	519904.21	4641461.55	x	x
M7	River	513889.78	4635948.32	x	x
V1	Sea	512810.13	4677223.23	x	x (a)
V2	Sea	516827.22	4676926.48	x	x
V3	Sea	528548.45	4684954.35	x	
V4	Sea	528531.27	4682275.27	x	x
V5	Sea	521445.12	4675618.12	x	x
V6	Sea	523829.98	4678175.33	x	
V7	River	519381.34	4672184.12	x	x
V8	River	517077.33	4674955.11	x	
V9	Sea	514940.17	4664660.25	x (c)	
	**Type**	**Population served (inh.)**		**24h composite sample**	
W1	Treated wastewater	150,000		x	
W2	Treated wastewater	7,000		x	
W3	Treated wastewater	25,000		x	
W4	Treated wastewater	17,200		x (c)	
W5	Treated wastewater	27,000		x (c)	

(a) POCIS lost in 1st campaign. (b) POCIS lost in 2nd campaign (C) Sampled only in 2nd campaign

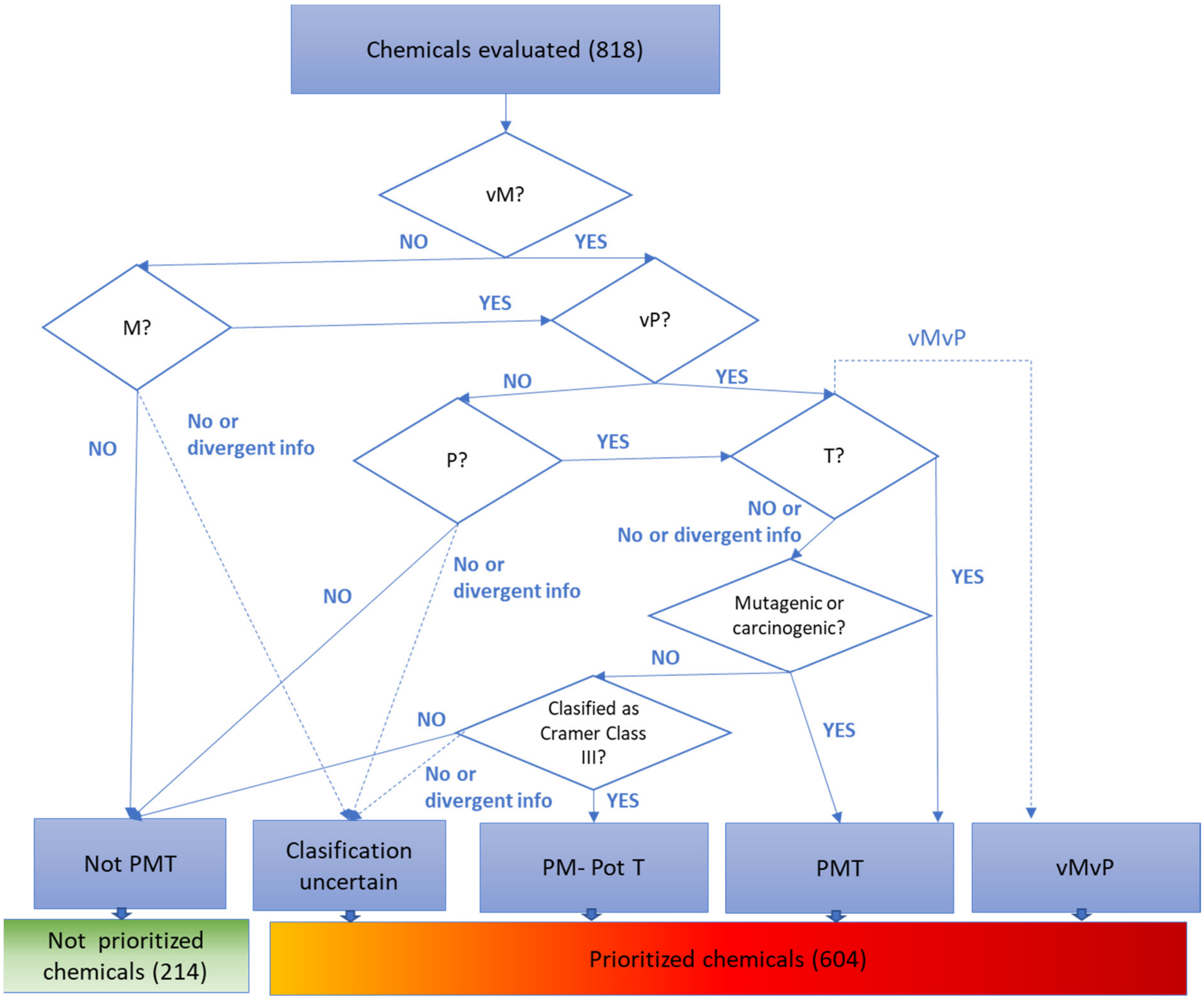

Figure S1: Decision tree for prioritization protocol

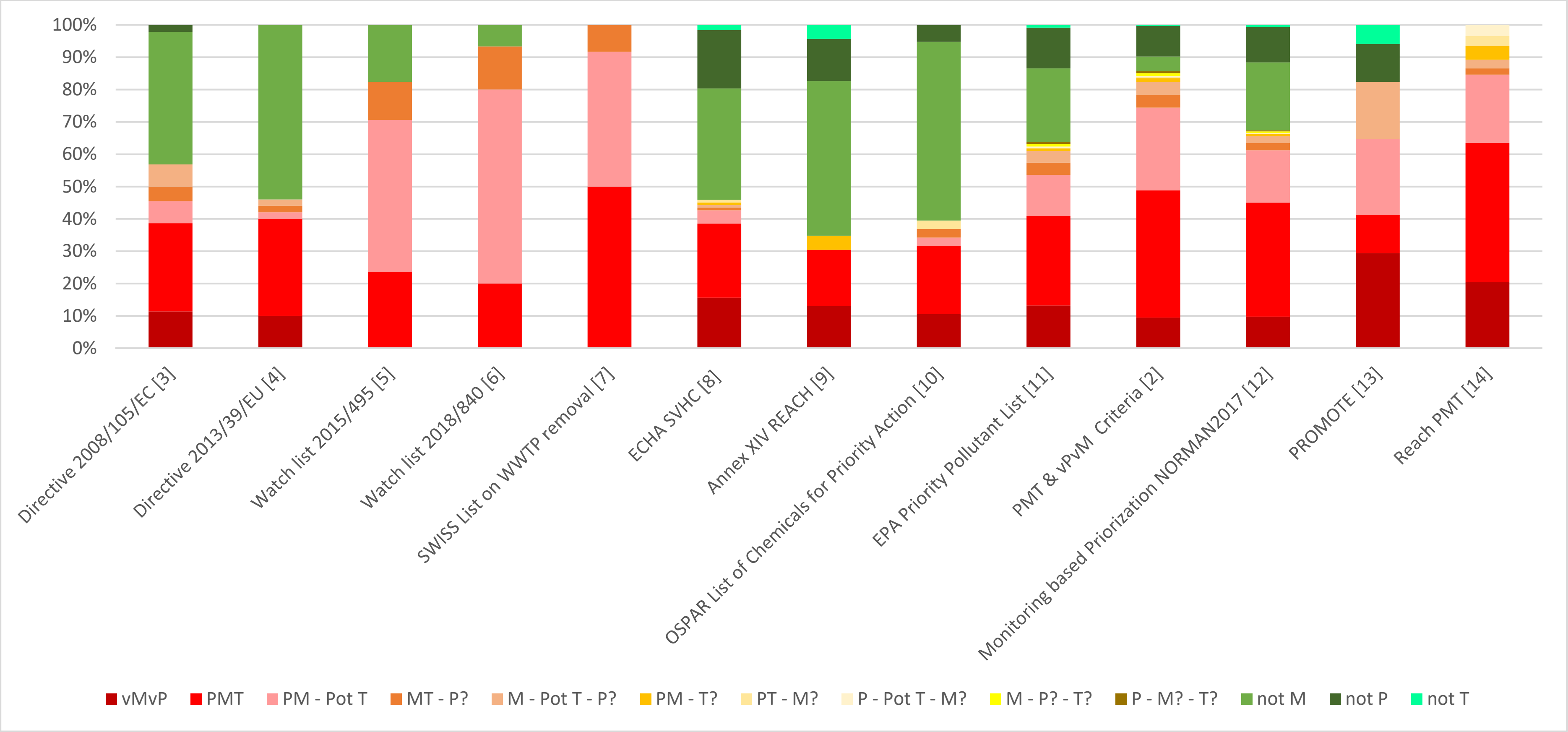

Figure S2: Classification of the substances contained in different sources of information according to this work's criteria

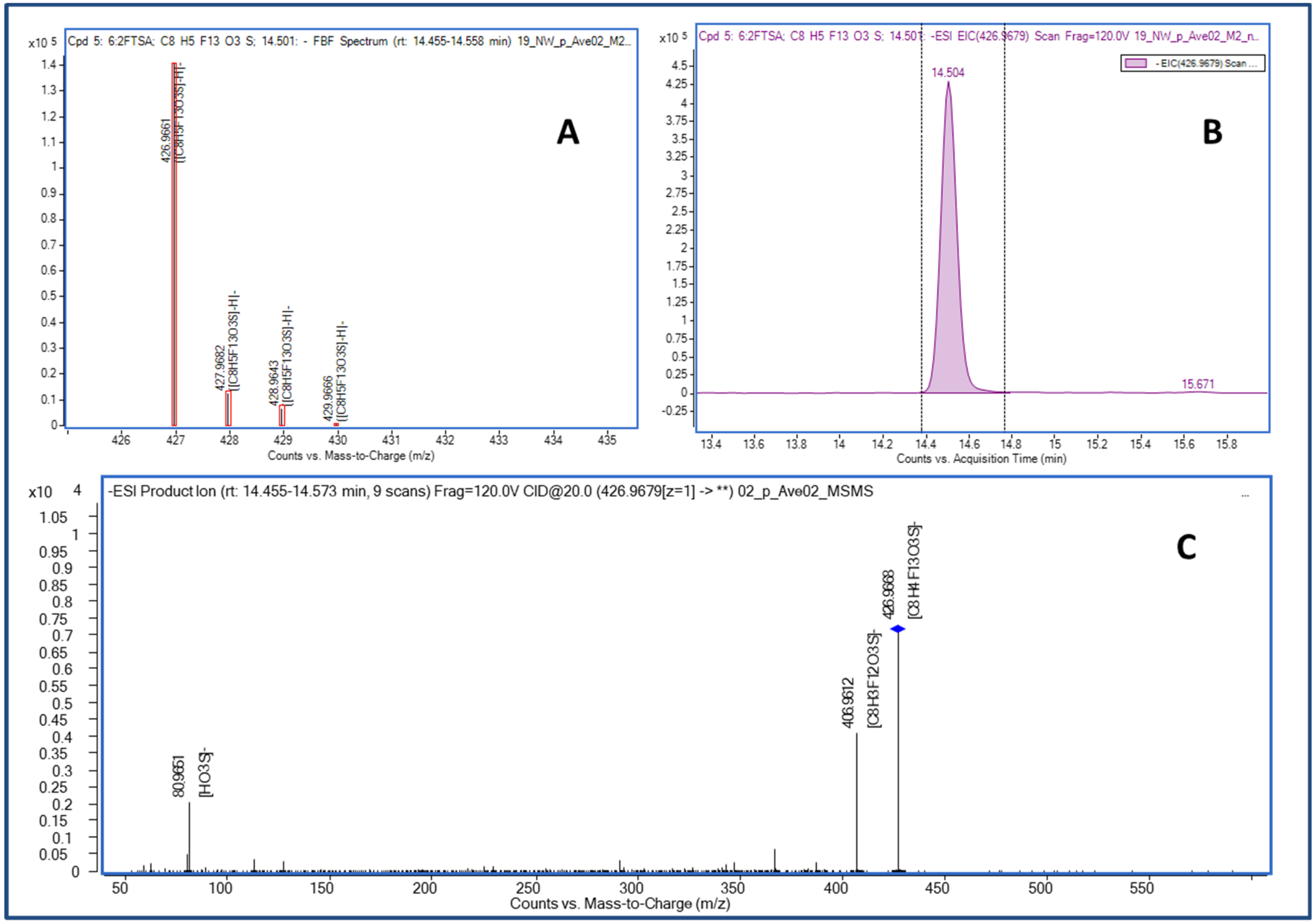

Figure S3: Identification of 6:2FTSA in sample A2. A) Match for the [M-H]⁻ isotopic distribution (theoretical values as red boxes), B) Extracted ion chromatogram of the [M-H]⁻ (*m/z* 426.9679) and C) experimental MS/MS spectrum at 20 V of collision energy, to be compared with recorded in MzCloud (reference number 8374).
